# Successful nucleofection of rat adipose-derived stroma cells with *Ambystoma mexicanum* epidermal lipoxygenase (AmbLOXe)

**DOI:** 10.1186/scrt503

**Published:** 2014-10-09

**Authors:** Angela Fülbier, Reinhild Schnabel, Stefanie Michael, Peter M Vogt, Sarah Strauß, Kerstin Reimers, Christine Radtke

**Affiliations:** Department of Plastic, Hand and Reconstructive Surgery, Hannover Medical School, Carl-Neuberg-Straße 1, 30625 Hannover, Germany

## Abstract

**Introduction:**

Adipose-derived stroma cells (ASCs) are attractive cells for cell-based gene therapy but are generally difficult to transfect. Nucleofection has proven to be an efficient method for transfection of primary cells. Therefore, we used this technique to transfect ASCs with a vector encoding for *Ambystoma mexicanum* epidermal lipoxygenase (AmbLOXe) which is a promising bioactive enzyme in regenerative processes. Thereby, we thought to even further increase the large regenerative potential of the ASCs.

**Methods:**

ASCs were isolated from the inguinal fat pad of Lewis rats and were subsequently transfected in passage 1 using Nucleofector^®^ 2b and the hMSC Nucleofector kit. Transfection efficiency was determined measuring co-transfected green fluorescent protein (GFP) in a flow cytometer and gene expression in transfected cells was detected by reverse transcription polymerase chain reaction (RT-PCR). Moreover, cell migration was assessed using a scratch assay and results were tested for statistical significance with ANOVA followed by Bonferroni’s post hoc test.

**Results:**

High initial transfection rates were achieved with an average of 79.8 ± 2.82% of GFP positive cells although longer cultivation periods reduced the number of positive cells to below 5% after four passages. Although successful production of AmbLOXe transcript could be proven the gene product had no measureable effect on cell migration.

**Conclusions:**

Our study demonstrates the feasibility of ASCs to serve as a vehicle of AmbLOXe transport for gene therapeutic purposes in regenerative medicine. One potential field of applications could be peripheral nerve injuries.

## Introduction

The limitations of human nerve regeneration often lead to unsatisfying results and impose special demands on reconstructive surgery. While smaller lesions might result in sufficient nerve regeneration, larger gaps practically cannot be bridged by the regrowing proximal nerve stump. Nerve regeneration depends on the structural and biochemical composition of the existing microenvironment and time is an important factor to maximize the positive outcome. Even when impairing factors are missing, supplementation of additional stimuli including substructural elements and messenger molecules such as growth factors enhance and direct axonal outgrowth [1]. Most promising approaches include cell and gene therapy to functionalize and optimize the microenvironment of the nerve growth cone [2, 3]. Gene therapy is mainly intended to enhance the velocity of axonal regeneration, which is important with regard to the increasing degeneration of the distal nerve stump and the resulting continuing loss of motor neurons [3]. Accordingly, most bioactive molecules that have been delivered to experimental models of denervated nerve stumps and injured spinal and facial motor neurons by gene transduction are neurotrophic proteins, although cell adhesion molecules are also feasible [3].

The Mexican axolotl (*Ambystoma mexicanum*) is an outstanding example of regenerative capacity and has the ability for regeneration of functional body structures like whole limbs as well as scar-free wound healing [4, 5]. The axolotl’s regenerative abilities also include severe injuries to the peripheral nerve system and the spinal cord [6, 7]. As there is constant interest to develop strategies allowing the transfer of this regenerative ability to mammals, we intended to identify factors that promote healing processes in the axolotl. In our previous study, we described the identification of an enzyme expressed in regenerating axolotl limbs which belongs to the epidermis-type of lipoxygenases (*A. mexicanum* epidermal lipoxygenase (AmbLOXe)) [8]. The lipoxygenases belong to the family of dioxygenases catalyzing the hydroperoxidation of polyunsaturated fatty acids using linolic acid and arachidonic acid as natural substrates. Lipoxygenase-initiated mediator pathways activate proinflammatory and anti-inflammatory signals alike [9], enabling a tight regulation of innate immunity and inflammation. AmbLOXe-expressing human cell populations showed increased rates of cell migration *in vitro*[8].

In this study we hypothesized that adipose-derived stroma cells (ASCs) can be nucleofected with a vector encoding for AmbLOXe to use them for cell-based gene therapy. A protocol for efficient transient transfection of ASCs was developed and used for the production of AmbLOXe-expressing cell populations. To test their biological efficiency, the generated ASC populations were kept in co-cultures with primary neurons.

## Materials and methods

### Adipose-derived stroma cell isolation and culture

ASCs were isolated using inguinal fat depots of adult male Lewis rats weighing 350 to 400 g. The animals were kept under standard conditions and written consent was obtained by the Animal Welfare Commissioner of the Hannover Medical School.

About 10 ml fat tissue was prepared from two anesthetized rats and the animals were sacrificed afterwards. The tissue was minced and digested under shaking by collagenase type I (CLS I, 2 mg/ml; Biochrom, Berlin, Germany) at 37°C for 60 minutes. The cell suspension was centrifuged at 175 × *g* for 5 minutes immediately after the digestion and after a washing step with Hank’s balanced salt solution (PAA, Pasching, Austria) +0.5% bovine serum albumin (Sigma-Aldrich, St. Louis, MO, USA). The resulting cell pellet was seeded in two 150 cm^2^ cell culture flasks (Biochrom) using Dulbecco’s modified Eagle’s medium/F12 medium (Biochrom) with 100 U/ml penicillin, 100 mg/ml streptomycin (PAA), 0.2 mM l-ascorbic acid-2-phosphate (A2P; Sigma), and 10% fetal bovine serum (Biochrom) and kept under standard cell culture conditions.

The cells were characterized following the suggestions of the International Society for Cellular Therapy [10]. In brief, the expression of surface markers on freshly isolated ASCs, passaged ASCs and nucleofected ASCs was determined by flow cytometry using CD11b/c PerCP-eFluor^®^ 710 (eBioscience, Frankfurt, Germany), CD44H-FITC (BD Bioscience, Heidelberg, Germany), CD45-FITC (Biolegend, Fell, Germany), CD90-PE/CY7 (Biolegend), CD34 (Santa Cruz, Heidelberg, Germany), and CD73 (BD Pharmingen™, Heidelberg, Germany). When needed, unconjugated primary antibodies were incubated with 1:10 diluted fluorochrome-labeled bovine anti-goat IgG-PerCP-Cy5.5 (Santa Cruz) or goat-anti-mouse-IgG-PE (Santa Cruz) as secondary antibodies. The labeled cells were analyzed by a FC500 flow cytometer (Beckman Coulter, Krefeld, Germany). Those measurements were done either immediately after the isolation, for selected samples after each passage or 7 days after the nucleofection process.

To survey the multipotency of the isolated cells they were kept under inducing conditions as described in the literature [11]. The cells were analyzed with the respective histological staining after the appropriate time.

### Nucleofection of adipose-derived stroma cells

AmbLOXe [GenBank:EU814616.1] derived from AmbLOXe pSTBlue-1plasmid [8] was subcloned into a unique *Eco*RI restriction site of the pIRES–EGFP vector (Clontech, Mountain View, CA, USA). The resulting plasmids were purified using the EndoFree Plasmid Maxi Kit (Qiagen, Hamburg, Germany). The fidelity and orientation of pIRES-EGFP-AmbLOXe (pI-AmbLOXe) was confirmed by restriction digest and sequencing at a local sequencing service (GATC, Stuttgart, Germany).

Cultures of ASCs were nucleofected at passage 1 with either pI-AmbLOXe, pIRES-EGFP (empty vector control), and/or the control vector pmaxGFP^®^ (Lonza, Basel, Switzerland) using the Human MSC Nucleofector kit (Lonza) according to the manufacturer’s instructions. Negative controls were transfected without adding vector DNA. In brief, 10^5^ cells were resuspended in 100 μl Nucleofector solution, mixed with 3 μg plasmid DNA, transferred to a cuvette and nucleofected using program A-033 of the nucleofector device. Control cells were treated analogously, but without DNA addition. Nucleofected samples were transferred to 500 μl prewarmed medium and seeded in six-well plates at a density of 10^5^ cells per well. Cells were incubated 3 days under standard conditions before being analyzed. The nucleofection efficiency was determined by detection of green fluorescent protein (GFP)-positive cells in a flow cytometer (FC-500; Beckman Coulter). To analyze the stability of nucleofection, the percentage of GFP-positive cells was measured on days 3, 6, and 12 after cell passage. Cells were subcultured four times at a ratio of 1:1, with 3 days between each plating.

### Comparison of transfection methods

Different transfection reagents were tested for their efficiency following the protocols provided by the manufacturers: FugeneHD (Promega, Madison, WI, USA), X-tremeGene 9 (Roche, Mannheim, Germany), and X-tremeGene-HP (Roche).

### Gene expression analysis

To confirm AmbLOXe expression in transfected ASCs, 500 ng total RNA were isolated by NucleoSpin^®^ RNA II kit (Macherey-Nagel, Düren, Germany) and reverse transcribed using the iScript cDNA Synthesis Kit (BioRad, Munich, Germany) according to the manufacturer’s instructions. Polymerase chain reactions were performed with AmbLOXe-forward primer 5′-ATGGTGGATGAGTACCGCATCAAAGA-3′ and AmbLOXe reverse primer 5′-TATGGACACACTGTTCTCTATCACTT-3′ using Advantage^®^ 2 Polymerase Mix (Clontech/Takara, Mountain View, CA, USA), Advantage™ 2SA buffer (Clontech/Takara), 2.5 μM nucleotide mixture (Clontech/Takara), and 2 μl produced cDNA on a Mastercycler Personal (Eppendorf, Hamburg, Germany). As a positive control, 0.5 μl AmbLOXe-pIRES vector was run in parallel. The amplification products were analyzed on a 1% (w/v) agarose gel supplemented with ethidium bromide and documented by the Biovision system (Vilber Lourmat, Eberhardzell, Germany).

### Cytotoxicity and cell metabolism

Cytotoxicity testing was done with CytoTox One (Promega) and determination of the cell metabolic activity with CellTiter-Blue (Promega).

Cells (10^3^) in 200 μl standard cell culture medium were seeded into 96-well plates in hexaplicate 30 minutes after nucleofection. The cells were incubated for 24 or 72 hours under standard cell culture conditions. Living ASCs without nucleofection and cells lysed in the lysis buffer included in the kit were used as the respective controls. Medium without cells was measured for determination of the background.

For the cytotoxicity assay, 100 μl cell culture supernatant were transferred to a new 96-well plate to proceed as stated by the manufacturer. For determination of the metabolic cell activity, substrate was directly added to the cultures and incubated at 37°C for 3 hours, following the instructions of the manufacturer.

Fluorescence was measured at excitation 560 nm/emission 590 nm on a filter-equipped plate reader (Genios, Tecan, Grödig, Austria). The measurements were repeated at two independent times. Means and standard deviations were calculated and tested for statistical significance with analysis of variance followed by Bonferroni’s *post hoc* test.

### Scratch assay

To analyze the cell migration, scratch tests were performed for ASC populations that either were left without nucleofection or were nucleofected without DNA or with pI-AmbLOXe, pmaxGFP^®^ or a mixture of both (1:2), respectively. The day after nucleofection, cells were seeded in triplicate into 12-well plates at 10^5^ cells per well and cultured under standard conditions (Dulbecco’s modified Eagle’s medium/F12 with 100 U/ml penicillin, 100 mg/ml streptomycin, 0.2 mM l-ascorbic acid-2-phosphate, and 10% fetal bovine serum, humidified atmosphere, 37°C, 5% carbon dioxide) for 2 days, in which time they reached confluence. The monolayers were then wounded with a disposable plastic pipette tip (10 to 100 μl volume) to create the scratch, rinsed with phosphate-buffered saline (Gibco, Grand Island, NY, USA), and incubated in ASC medium. The scratches were documented microphotographically every 6 hours over 36 hours. Closing of scratches was analyzed using the image analysis software Cell-D (Olympus, Hamburg, Germany). The scratch length and cell-free area were measured and the mean distances between cell fronts were calculated. Scratch tests were carried out in four independent experiments. Means and standard deviations were calculated and tested for statistical significance with analysis of variance followed by Bonferroni’s *post hoc* test.

### Isolation of neurons from dorsal root ganglia

Dorsal root ganglia were excised from adult rats previously sacrificed under anesthesia in ice-cold conditions. The collected ganglia were prepared and digested with 1.7 mg/ml collagenase A (Roche), 1.7 mg/ml collagenase D (Roche), and 1.25 mM calcium chloride (Sigma). An additional treatment with 2.5 mg/ml papain (Sigma), 100 mM l-cystein (Sigma) and 10 mM ethylenediamine tetraacetic acid (Sigma) was performed at 37°C for 5 minutes. After mechanical dissociation the neurons were kept in a modified Bottenstein and Sato medium consisting of Dulbecco’s modified Eagle’s medium/F12 + 6% d-glucose (Sigma) supplemented with 100 μg/ml bovine serum albumin, 100 μg/ml transferring (Sigma), 100 μM putrescine (Sigma), 30 nM sodium selenite (Sigma), 20 nM progesterone (Sigma), 10 nM insulin (Sigma), and 1% of 100 U/ml penicillin, 100 mg/ml streptomycin and were plated onto laminin-coated (Sigma) cell culture vessels.

For co-culture experiments, the transfected ASCs were mixed with a freshly prepared suspension of dorsal root ganglia neurons and plated in triplicate at a density of 10,000 ASCs and 1,000 neurons on cover glasses. After 24 hours and 48 hours, respectively, the samples were fixed in 4% (w/v) paraformaldehyde (Karlsruhe, Germany) in phosphate-buffered saline and analyzed by digital microphotography using the CKX41 imaging system (Olympus) and image analysis software imageJ (National Institutes of Health, Bethesda, MD, USA). The numbers and lengths of the outgrowing neurites were measured in five independent experiments. The results are presented as means and standard deviations and were tested for statistical significance with Kruskal–Wallis analysis of variance followed by Bonferroni-corrected Mann–Whitney *U* test.

## Results

### Nucleofection of adipose-derived stroma cells

AmbLOXe, an effector of amphibian regeneration, was shown to induce increased *in vitro* wound closure rates in some mammalian cell lines that were transfected with it [8]. Aiming at an improvement in tissue regeneration, AmbLOXe included in pIRES-EGFP vector (pl-AmbLOXe) was nucleofected into rat ASCs.

Our pre-examinations to select a suitable transfection technique revealed that the mean transfection efficiencies were very low with common transfection reagents, such as FugeneHD (0.21 to 2.2%), X-tremeGene 9 (0.0 to 0.05%), and X-tremeGene-HP (0.06 to 0.3%), with slight variances depending on the relation between DNA and reagent.

**Figure 1:**
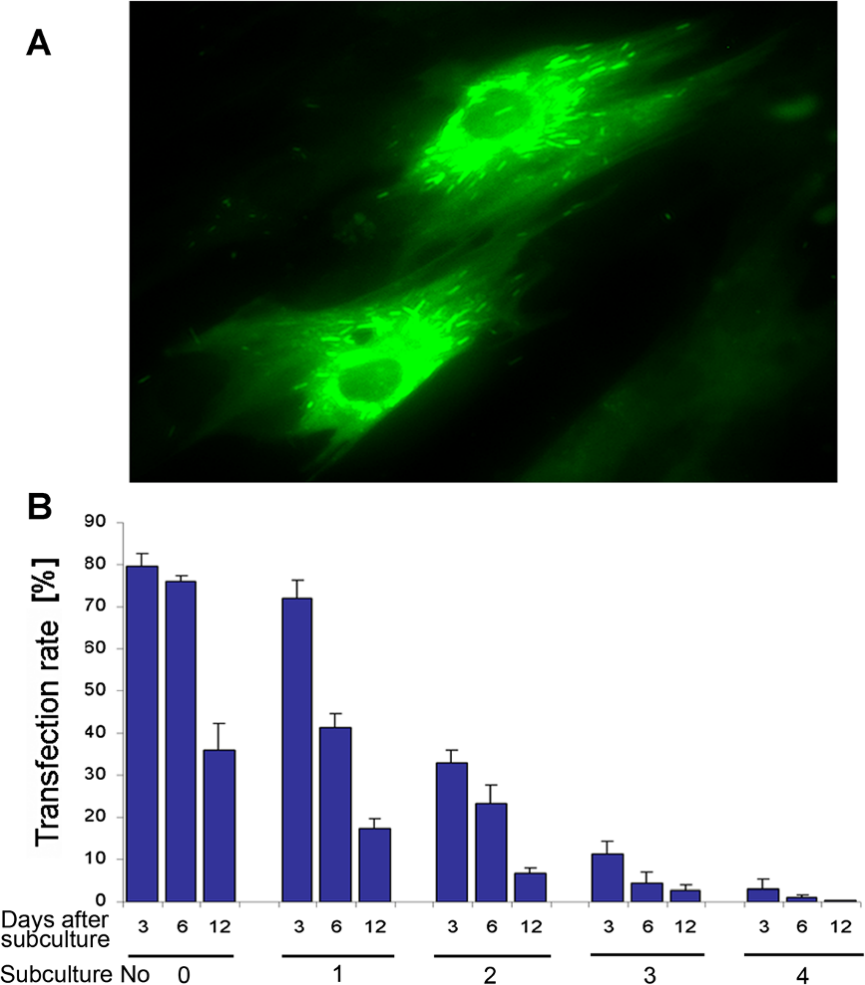
**Nucleofection of adipose-derived stroma cells.** For the establishment of a transfection protocol, the vector pmaxGFP^®^ encoding for green fluorescent protein (GFP) was used and detected with a fluorescent microscope **(A)** or a flow cytometer **(B)**. Data are presented as percentages of GFP-positive cells. The transfection rates were determined to be 79.8 ± 2.82% on average at the beginning but showed reducing numbers of positive cells over time and passage. (B) First three columns represent the original culture after the nucleofection without subculturing (subculture No. 0); following columns are grouped as passages 1 to 4 after nucleofection as indicated below (subculture No. 1 to 4). The numbers directly under the columns indicate the days after nucleofection or passaging, respectively.

In contrast, ASCs could successfully be transfected by nucleofection. The efficiency of this technique was determined by the control vector pmaxGFP^®^, resulting in bright green fluorescence after vector uptake (Figure 1A). The percentages of positive cells were analyzed by subsequent flow cytometry. The mean transfection efficiency obtained at day 3 after nucleofection (maximum of expression) was 79.8 ± 2.82% (*n* =11). The nucleofection was highly reproducible but transient; 12 days after the gene transfer, only 36.05 ± 6.29% of cells expressed GFP. When the nucleofected cells were subcultured, the average percentage of GFP-positive cells was 3.03 ± 2.4% after four passages (Figure 1B).

### Detection of AmbLOXe expression

**Figure 2:**
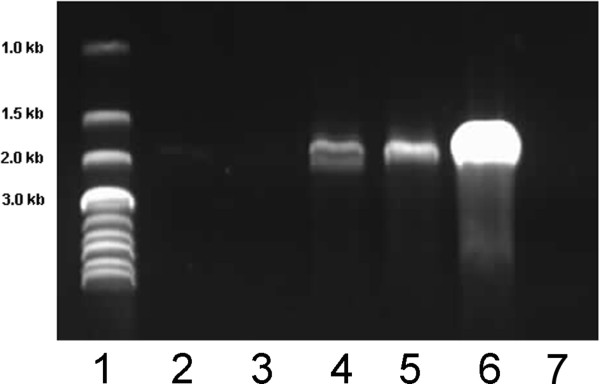
**Expression of AmbLOXe in the nucleofected adipose-derived stroma cells** ***.*** *Ambystoma mexicanum* epidermal lipoxygenase (AmbLOXe) expression was detected by polymerase chain reaction in the nucleofected cell populations 5 days after transfection. In pIRES-EGFP-AmbLOXe (pI-AmbLOXe) nucleofected cells, pI-AmbLOXe/pmaxGFP^®^ co-nucleofected cells (ratio 1:1) as well as in the positive control (pI-AmbLOXe vector), a band corresponding to the size of full-length AmbLOXe could be detected (lanes 4 to 6). No bands were observed after nucleofection without DNA or with pmaxGFP^®^ alone (lanes 2 and 3). Lane 1, DNA ladder; lane 2, nucleofection without DNA; lane 3, nucleofection with pmaxGFP^®^; lane 4, nucleofection with pI-AmbLOXe; lane 5, nucleofection with pI-AmbLOXe/pmaxGFP^®^; lane 6, pI-AmbLOXe alone as vector (positive control); lane 7, water (negative control).

AmbLOXe expression was detected by polymerase chain reaction amplification of cDNA reverse transcribed from RNA isolated from nucleofected cell populations. The nucleofection with pI-AmbLOXe was compared with nucleofection with pmaxGFP^®^, a co-nucleofection of pI-AmbLOXe/pmaxGFP^®^, as well as a control nucleofection without DNA (Figure 2). As a positive control, the same polymerase chain reaction was performed with diluted vector encoding AmbLOXe.

In samples nucleofected with pI-AmbLOXe and in the positive control, a band around 1,800 base pairs – which corresponds to the size of full-length AmbLOXe – could be detected (Figure 2, lanes 4 to 6). No bands were observed after nucleofection without DNA or with pmaxGFP^®^ (Figure 2, lanes 2 and 3, respectively).

### Lactate dehydrogenase release and cell metabolic activity

**Figure 3:**
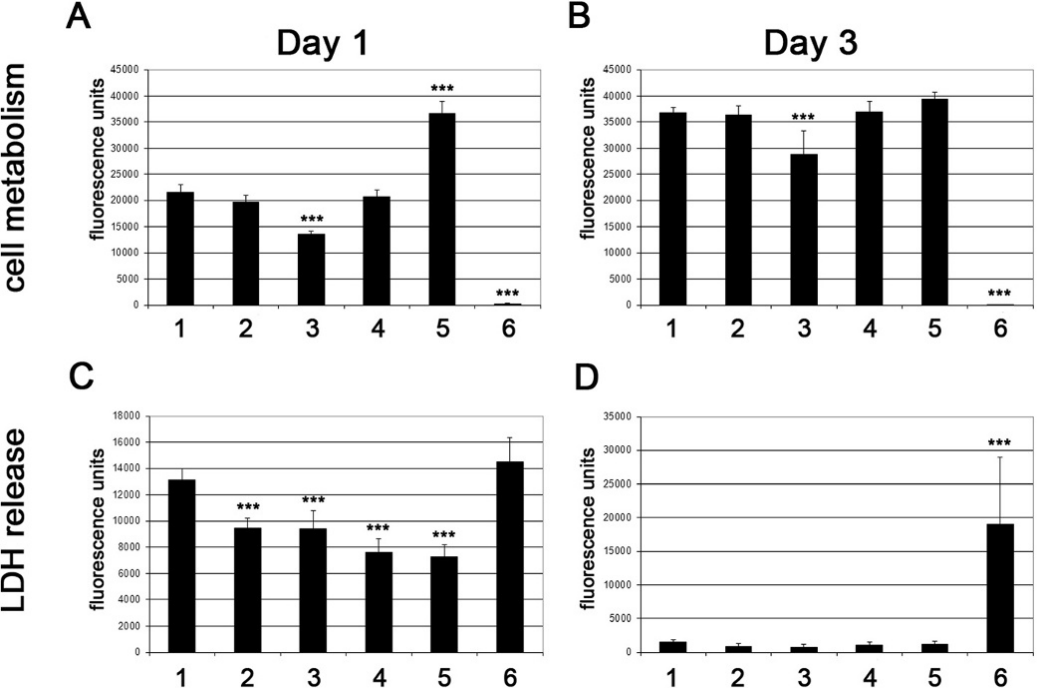
**Measurement of lactate dehydrogenase release and cell metabolism** ***.*** Cells were seeded into 96-well plates and incubated for the indicated time points. The respective substrates were either added to the cells (cell metabolism) or to the cell culture supernatants (lactate dehydrogenase (LDH)). After the appropriate time and conditions, fluorescence was measured and given in arbitrary units. **(A), (B)** Cell metabolism on day 1 and day 3, respectively. **(C)**, **(D)** LDH release on day 1 and day 3, respectively. Nontransfected cells (columns 5 and 6) were used as the respective controls. Significant differences observed between the *Ambystoma mexicanum* epidermal lipoxygenase (AmbLOXe)-expressing cells and the other cell populations are indicated (***P >0.001). Group 1, pIRES-EGFP-AmbLOXe (pI-AmbLOXe); Group 2, pmaxGFP^®^; Group 3, 50% pI-AmbLOXe and 50% pmaxGFP^®^; Group 4, cells nucleofected without DNA; Group 5, untreated adipose-derived stroma cells (ASCs); Group 6, untreated ASCs in lysis buffer.

To rule out any harmful effect of nucleofection and/or AmbLOXe expression, we measured lactate dehydrogenase (LDH) release and resazurin to resorufin reduction by cell metabolism (Figure 3). On day 1 after nucleofection, all samples that underwent the procedure showed reduced cell metabolism compared with the untreated cells (Figure 3A). On day 3 most cell populations recovered from the process, with exception of the double-transfected cells (pApmax) (Figure 3B). The first day after handling the cells, LDH release was measurable in all cell populations, especially those expressing AmbLOXe (Figure 3C), but on day 3 all cell populations recovered and only trace amounts of LDH could be detected (Figure 3D).

### Influence of AmbLOXe expression on the ASC phenotype

The cell surface markers of mesenchymal stem cells (MSCs) as assigned by Dominici and colleagues were determined before and 7 days after nucleofection [10]. No significant difference was observed in cell populations that were transfected with a vector encoding for AmbLOXe or with empty vectors, and in cells transfected without adding DNA or left completely untreated (data not shown).

**Figure 4:**
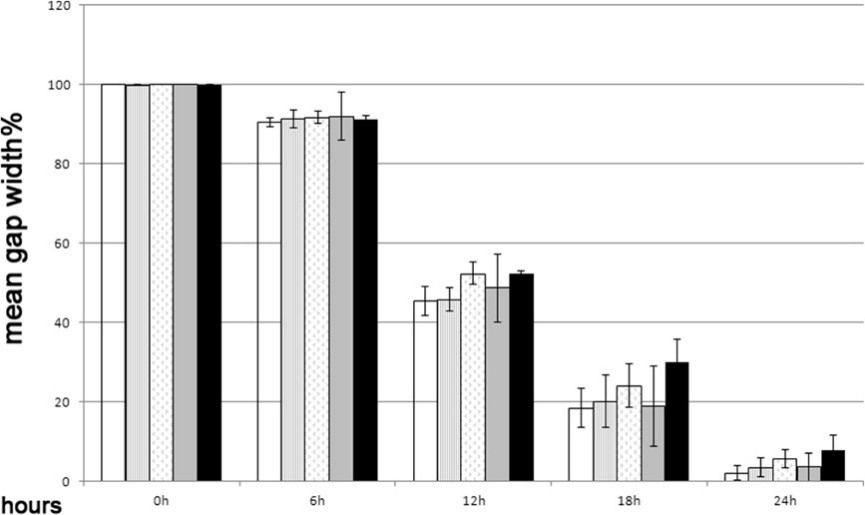
**Scratch test of nucleofected adipose-derived stroma cells** ***.*** Scratch tests in confluent monolayers of adipose-derived stroma cells (ASCs) nucleofected with pIRES-EGFP-AmbLOXe (pI-AmbLOXe), the empty vector or a mixture of both. No significant differences between the groups could be found over time. White column, cells nucleofected without DNA; striped column, pI-AmbLOXe; patterned column (small arrowheads), 50% pI-AmbLOXe and 50% pmaxGFP^®^; grey column, pmaxGFP^®^; black column, untreated ASCs. AmbLOXe, *Ambystoma mexicanum* epidermal lipoxygenase.

To determine whether AmbLOXe expression had a measurable influence on ASC migration velocity, confluent monolayers of ASCs nucleofected without DNA, with pI-AmbLOXe, with a GFP-expressing vector (pmaxGFP^®^) or with a mixture of both (50% pI-AmbLOXe and 50% pmaxGFP^®^), were scratched and their closing analyzed. In all groups, scratches were closed after 30 hours. Untreated ASCs were used as controls. There was a uniform decrease of mean distance, and no significant difference between the groups could be found over time (Figure 4).

### Influence on number and length of outgrowing neurites in co-culture experiments

**Figure 5:**
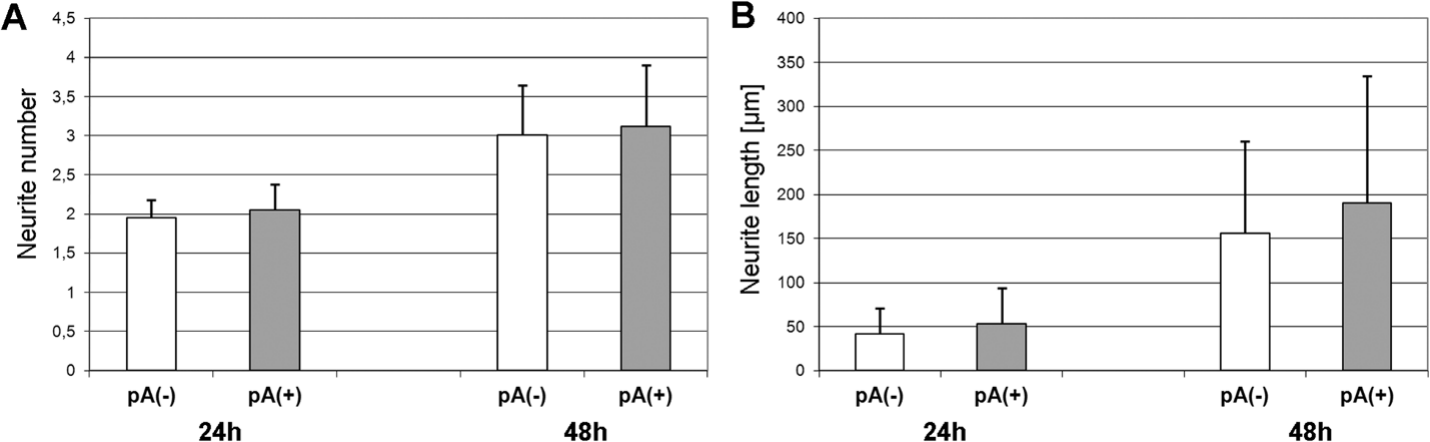
**Morphometric analysis of neurite numbers and lengths of neurons kept in co-cultures with adipose-derived stroma cells. (A)** Mean number of neurites in absolute numbers. **(B)** Mean length of neurites (μm). Time points of data analyses were after 24 hours and 48 hours, respectively. White column, co-cultures without AmbLOXe-expressing ASCs (pA(-)); grey column, co-cultures with AmbLOXe-expressing ASCs (pA(+)). AmbLOXe, *Ambystoma mexicanum* epidermal lipoxygenase; ASC, adipose-derived stroma cell.

To test whether ASCs could be used for cell-based gene therapy delivering ectopically expressed AmbLOXe, we next performed co-culture experiments. Primary neurons derived from rat dorsal root ganglions and ASC cell populations transfected with either a vector encoding for AmbLOXe or with the same vector without coding sequence were kept together for 24 or 48 hours, respectively. Counting the mean number of outgrowing neurites revealed no difference between the samples, neither after 24 hours nor after 48 hours. Concerning the neurite length, a slight difference could be detected. After 24 hours, the mean length of neurites was 41.3 ± 29.18 μm when the neurons were kept in co-cultures with an ASC population nucleofected with the empty vector and was 53.27 ± 40.96 μm when the co-cultures contained AmbLOXe-expressing ASCs. After 48 hours the mean lengths were 155.63 ± 104.98 μm compared with 190.49 ± 143.87 μm (Figure 5). Although the differences were not significant due to the high variances of neurite lengths, there is a tendency for AmbLOXe expression in ASC populations to enhance neurite outgrowth.

## Discussion

Adult MSCs are a widely used cell type in regenerative medicine. There are a number of studies indicating that transplanted MSCs are attracted to sites of tumors/metastases, injury, inflammation and ischemia [12]. In the setting of stem cell transplantation, MSCs from neonatal skin have been shown to be a potential therapy for collagen VI-related congenital muscular dystrophy by integration of the cells into the skeletal muscle and production of the missing collagen VI in a mouse model [13]. Moreover, MSCs seem to be an effective therapy for autoimmune encephalomyelitis and multiple sclerosis, also in a mouse model [14]. A combination of stem cell therapy and myocardial gene therapy in the peri-infarcted region of rats improved cardiac function 4 weeks after myocardial infarction [15]. ASCs can also be used for cell-based gene therapy by overexpressing the desired therapeutic factors; for example, for enhancing angiogenesis [16]. ASCs have been defined as a subpopulation of adipose tissue stroma cells with a multipotent differentiation capability and are thus regarded as a special type of MSCs [11]. ASCs are able to integrate into host tissue and to differentiate into many different tissue types. Further, ASCs are a convenient source of adult stem cells since they can be isolated from lipoaspirates with minimal invasive surgical intervention, they can be harvested in sufficient amounts, and they are easy to propagate in cell culture [11]. These observations emphasize the versatility of MSCs/ASCs in different regenerative processes, possibly combined with gene therapy.

Exploitation of MSCs/ASCs as vehicles for cell-based therapies is feasible but is hampered by the relatively weak transfectability of these primary cells [16]. We and others tried transfection reagents with limited success; for example, Aluigi and colleagues used nucleofection successfully to transfect human MSCs, in contrast to commercial transfection reagents such as Fugene and DOTAP that transfected the cells at the low rate of 3.6 ± 2.4% and 5.4 ± 3.4% respectively [17]. The same was observed in other studies where treatment of human MSCs with various transfection reagents resulted in low transfection rates or no transfection at all [18, 19].

The use of viral vectors has been proposed [20] but falls under safety concerns [21] and offers limited space for foreign gene integration. Other methods used for MSC/ASC transfection include chemical and physical methods such as polyethyleneimine-mediated gene delivery [22], biodegradable polymeric vectors [23], or electroporation [24]. In our study we used the nucleofection method for transient transfection of rat ASCs at high rates. Nucleofection is a method to deliver plasmid DNA into cells based on an electroporation system equipped with programs specific for different cell types. It is possible to vary the duration, frequency and voltage of the electrical pulses in combination with cell type-specific transfection buffers. This has been used successfully for a number of cells difficult to transfect, among them human bone marrow-derived MSCs [25]. Although the original protocol was designed for human stem cell transfection, we could adapt it to the isolated rat ASCs. When tested with a GFP-encoding vector, transfection rates of 79.8 ± 2.82% could be achieved and AmbLOXe transcript could be demonstrated in ASC populations transfected with an AmbLOXe-encoding vector. Although the cells were affected by the handling and the nucleofection, they quickly recovered to normal cell metabolism and LDH release rates on day 3.

To rule out any influence of AmbLOXe expression on the ASCs, we detected MSC markers on the surface of cell populations either expressing AmbLOXe or not. No difference concerning these marker proteins’ expression could be observed. We next performed scratch assays to assess the migration velocity, which is a clear sign of AmbLOXe activity [8], but no influence on the ASCs respective to the controls could be observed. As no obvious change in the ASC phenotype could be detected, we conclude that ASCs can be used as vehicles for the transport of AmbLOXe-encoding vectors without themselves being affected.

The regenerative potential of both the ASCs as such and the addition of AmbLOXe might be used in clinical settings such as healing of damaged nerves. In the case of peripheral nerve injury, an early stimulation of neurite outgrowth is favorable and long cell extrusions with minimal branches indicate directed outgrowth. This can be achieved by genetic transfer of neurotrophic factors such as nerve growth factor. The reaction of neurons to such growth factors were shown to be dosage dependent in a way that lower levels of transfection can be more effective than maximum levels [26]. In a first proof of principle, AmbLOXe-expressing ASC populations were able to stimulate neurite outgrowth in our direct co-culture experiments. Although the results were not significant, they were promising and require optimization. Possible steps to further enhance the biological effect of AmbLOXe-based gene therapy include the variation of the vector system; for example, exchanging the viral promoter with a strong cellular promoter. Also, the most effective dose of AmbLOXE-expressing cells needs to be determined. Therapeutic delivery of a lipoxygenase in a therapeutic context is a new approach. In recent studies, gene transfer of lipoxygenase 15-1 was used for inhibition of angiogenesis in different setting [27, 28]. We expect that genetic transfer of AmbLOXe as an inducer of cellular growth is an important option for future therapeutic approaches, especially in the context of nerve regeneration.

## Conclusion

ASCs can successfully and efficiently be transfected by nucleofection and thereby may serve as a vehicle for gene therapy. Especially, transfection with AmbLOXe may be valuable in the field of regenerative medicine; for example, in the case of peripheral nerve injuries.

## Abbreviations

AmbLOXe: *Ambystoma mexicanum* epidermal lipoxygenase
ASC: adipose-derived stroma cell
GFP: green fluorescent protein
LDH: lactate dehydrogenase
MSC: mesenchymal stem cell.
